# The Burden of Serious Fungal Infections in Kyrgyzstan

**DOI:** 10.3390/jof5030066

**Published:** 2019-07-19

**Authors:** Gulnura K. Turdumambetova, Ali Osmanov, David W. Denning

**Affiliations:** 1 Department of Phthisiology, Kyrgyz State Medical Academy, Bishkek 720020, Kyrgyzstan; 2 Global Action Fund for Fungal Infections, 1208 Geneva, Switzerland; 3 Faculty of Biology, Medicine and Health, University of Manchester, Manchester Academic Health Science Centre, Manchester M13 9NT, UK; 4 National Aspergillosis Centre, Wythenshawe Hospital, Manchester University NHS Foundation Trust, Manchester Academic Health Science Centre, Southmoor Road, Manchester M23 9LT, UK

**Keywords:** Kyrgyzstan, fungal infection, aspergillosis, candidiasis, epidemiology, cryptococcal meningitis, *Pneumocystis* pneumonia

## Abstract

Kyrgyzstan in Central Asia has a population of 6 million people who have high mortality rates for chronic lung diseases. The mountainous geography, widespread use of biomass fuels for cooking and indoor heating, and high rates of smoking are the major contributing factors. We have estimated the number of serious fungal infections in order to define the burden of these diseases in Kyrgyzstan. We estimated 774 cases of chronic pulmonary aspergillosis (CPA) as a sequel of tuberculosis (TB); CPA occurs as a sequel of multiple conditions, so a total prevalence of 3097 cases was estimated, which is among the highest rates in the world. An estimated 2205 patients have allergic bronchopulmonary aspergillosis (ABPA) and 2911 have severe asthma with fungal sensitization (SAFS), which may be an underestimate. There are approximately 292 cases of invasive aspergillosis annually. The number of adult women who get recurrent vulvovaginal candidiasis is 175,949. We approximated 787 cases of oral and 294 cases of esophageal candidiasis, 25 cases of cryptococcal meningitis, and 101 cases of *Pneumocystis* pneumonia annually in HIV-positive patients. The incidence of candidemia was estimated at 300. We have estimated that a total of 185,961 people (3% of the population) have serious fungal infection in Kyrgyzstan. Given this burden, diagnostic improvements are necessary.

## 1. Introduction

Serious fungal infections complicate many other disorders, affecting those with cancer, AIDS, and chronic respiratory disease, as well as critically ill hospitalized patients [1,2]. Occasional cases occur in apparently normal individuals, notably implantation mycoses such as mycetoma and fungal keratitis, as well as many cutaneous fungal infections [3]. Poor hospital infection control procedures and antibiotic abuse increase the risk of life-threatening invasive candidiasis. Immunocompromised patients are at particular risk, the risk varying by underlying condition and treatment given, and newly introduced therapies have increased this risk for some patients [4].

Even in countries with developed economies, there are limited epidemiological studies (besides those on candidemia) that focus on serious fungal infection [5,6]. As a result, there is a lack of awareness of this problem from most healthcare authorities. This hinders better understanding of the problem due to the insufficient allocation of resources. It was estimated that only 1.4%–2.5% of the infection and immunology research resources of major funding bodies are allocated to invasive fungal infections [7], and this research inequity is greater in middle- and low-income countries [8].

At the same time, the surveillance of fungal infection is neglected even in countries with developed healthcare systems [7]. To address this issue, the Leading International Fungal Education (LIFE) started an initiative to improve understanding of the scale of the burden of serious fungal infections globally and in individual countries. This work aims to evaluate the scale of the burden of serious fungal infections in Kyrgyzstan.

**Figure 1:**
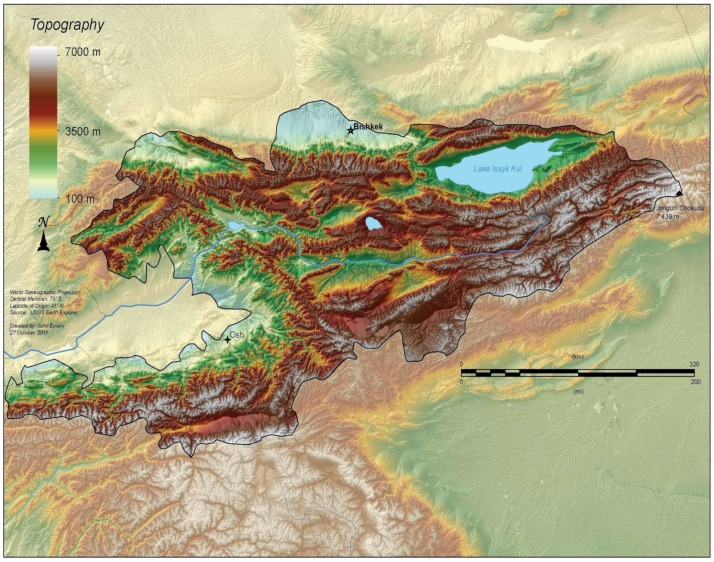
Geography of Kyrgyzstan; 90% of the area are highlands [12].

Kyrgyzstan is a low-income country in Middle Asia with a population of 6 million people, a GDP per capita of US$1070 [9], and high morbidity and mortality rates for chronic lung diseases of 9896.73/100,000 and 31.8/100,000, respectively, according to Kyrgyz Ministry of Health data [10]. Several factors contribute to high mortality: the mountainous geography (Figure 1), widespread use of biomass fuels for cooking and indoor heating, and high rates of smoking are the major contributing factors. According to Полупанов et al. [11], the smoking prevalence among both sexes is 25.6%, while 5% of Kyrgyz people are ex-smokers. Smoking rates among men are considerably higher than among women (being 46.6% and 8%, respectively); the mean number of smoked cigarettes per day is 12.7 (17.5 and 13.5 among men and women, respectively). Smoking is a factor that leads to the increased prevalence of lung diseases (chronic obstructive pulmonary diseases (COPD) and lung cancer, in particular) that are an underlying factor for the development of pulmonary fungal infection. Due to poor access for rural and nomadic people (who comprise more than 65% of the population) to healthcare facilities, the burden of respiratory diseases is highly likely to be significantly underestimated.

## 2. Materials and Results

To estimate the burden of fungal infection in Kyrgyzstan, we identified all epidemiology papers on fungal infection in English, Kyrgyz, and Russian languages. We searched several databases, namely, PubMed, Google Scholar, elibrary.ru, and Cyberleninka. The search terms were “fungal infection”, “epidemiology”, and each specific infection (e.g., “chronic pulmonary aspergillosis”). PubMed supports only English queries, while other databases were searched using the same queries in three languages: English, Russian, and Kyrgyz. The search covered all dates up to June 2019. However, we found no published data on this topic. As a result, we utilized the model proposed by LIFE [13]. The total burden of serious fungal infections, the rate per 100,000 people, is summarized in Table 1.

Despite the urgency of the problem of chronic pulmonary disease, there are no precise data on this topic. Estimates for the prevalence of COPD vary both in Kyrgyzstan [14] and for this region [15]. Hence, we used a median estimate of 14% of the over 30 age group, which resulted in 378,000 patients with COPD. Approximately 5% of patients with COPD are hospitalized once a year [16], which results in 18,900 hospital admissions annually. Invasive aspergillosis occurs in 1.3% of hospitalized COPD patients [17], so we approximated 246 patients with invasive aspergillosis (IA). However, due to the aforementioned reasons, the number of COPD admissions is probably higher in Kyrgyzstan, and in southern China, the IA incidence in COPD admissions was 3.9% [18], so our estimates are conservative. The prevalence of asthma in Kyrgyzstan is not known, but we have applied the results of To et al. [19], suggesting a 1.47% asthma prevalence in adults in neighbouring Kazakhstan. Allergic bronchopulmonary aspergillosis (ABPA) occurs in approximately 2.5% of asthma patients; hence, there are approximately 2205 patients with ABPA in Kyrgyzstan [20]. To estimate patients with severe asthma with fungal sensitization (SAFS), we assumed a 33% sensitization prevalence in adults with severe asthma (10% of total) and estimated 2911 patients with SAFS [21]. Unfortunately, fungal sensitization rates are not known for any country in Central Asia apart from Iran, where 19.6% of all adult asthmatic patients are sensitized to one or more fungi [22].

According to WHO data, there were 7695 cases of tuberculosis (TB) and 5848 cases of pulmonary TB in Kyrgyzstan. In accordance with previously published data, chronic pulmonary aspergillosis (CPA) occurs in 13%–23% of pulmonary TB. We assumed 15% annual mortality and a 6% resection rate, so we approximated 774 cases of CPA as a sequel of TB [23]. Due to the fact that CPA occurs as a sequel of multiple pulmonary conditions such as COPD, pneumothorax, sarcoidosis, and emphysema [20], and based on clinical data from Manchester [24], the prevalence of CPA in other conditions is three times higher than the prevalence of CPA as a sequel of TB. Thus, there are approximately 3097 cases of CPA in Kyrgyzstan, which is a very high rate of 51.6/100,000 [24].

The five-year prevalence of all forms of cancer is 12,407, while there are 672 cases of lung cancer annually [25]. We estimated 40 patients with IA in this group based on the 2.6% rate documented by Yan et al. in China [26]. The rate of invasive aspergillosis in acute myeloid leukemia (AML) (10%) [27] is equal to all other forms of leukemia, and applying the AML annual incidence of 4.7/100,000 (Globocan) means that there are a predicted six patients with IA among patients with hematological malignancies [28].

The number of HIV-positive patients in Kyrgyzstan is 8500 and only 28% currently have access to antiretroviral therapy, [29] Oral candidiasis occurs in 90% of patients with CD4 < 200 cells/mL, while esophageal candidiasis occurs in at least 20% of patients with CD4 < 200 cells/mL and in 5% of patients receiving antiretroviral therapy [30,31,32]. So, we estimated 787 cases of oral and 294 cases of esophageal candidiasis annually. Assuming a prevalence of cryptococcal antigenemia of 2.9% [33] and the number of patients with CD4 < 200 cells/ml to decline over seven years among patients not receiving antiretrovirals (ARVs), we estimated 25 cases of cryptococcal meningitis annually. In advanced HIV disease, we anticipate an annual incidence of *Pneumocystis* pneumonia (PCP) of 101 cases annually, which omits all other causes of the lethal infection [33]. PCP is partially preventable with prophylaxis, but it is not clear to what extent this is used in Kyrgyzstan in all patient groups.

We identified that the number of women between 15 and 50 years who are at risk for recurrent vulvovaginal candidiasis (rVVC) to be 2,932,484. Based on anonymous Internet surveys in Europe and the United States, the rate of rVVC is >6%, so we estimated 175,949 Kyrgyz women to be suffering from rVVC [34,35].

There are no data on candidemia in patients on peritoneal dialysis, so we used French data suggesting one case of postsurgical *Candida* peritonitis/intra-abdominal for every two patients with candidemia occurring in ICUs [36]. We used a low European average of 5.0 per 100,000 to estimate 300 patients with candidemia. As approximately 30% of these cases occur in ICUs [37], there are approximately 100 cases of candidemia occurring in ICUs. As a result, we estimated 50 cases of *Candida* peritonitis. This estimation ignores continuous ambulatory peritoneal dialysis (CAPD) peritonitis, as we do not have these figures for renal failure and dialysis.

We were not able to estimate the number of patients suffering from fungal keratitis and tinea capitis due to the absence of local data or information on the population at risk for these infections.

## 3. Discussion

In this study, we found that fungal infections affect 185,961 of Kyrgyz people, which comprises 3% of the population. The burden may be underestimated due to the lack of reporting and inaccurate records. *Candida* bloodstream infection underestimates invasive candidiasis; only 40% of the latter have a blood culture that is positive. So, if we are correct that there are about 250 cases of candidemia in Kyrgyzstan, there are probably 300 invasive candidiasis cases, including those with peritoneal (intra-abdominal) candidiasis. There are no reports of the species distribution of *Candida* in Kyrgyzstan and no knowledge about whether *Candida auris* has arrived there or not yet.

If untreated, *Candida* peritonitis, candidemia, cryptococcosis, PCP, and invasive aspergillosis are almost uniformly fatal infections, with very few exceptions. Chronic pulmonary aspergillosis is a slowly progressing disease that has a mortality rate of 25% within the first six months and 15% thereafter [38]. In Kyrgyzstan, the population burden of chronic pulmonary aspergillosis is among the highest in the world.

At the same time, there almost no fungal diagnostics capabilities in Kyrgyzstan other than culture; fungal culture is not very sensitive, and less so if samples are plated on bacterial media [22]. Essential antifungals, namely, flucytosine, liposomal forms of amphotericin, itraconazole, voriconazole, and caspofungin, are not available either; only fluconazole and terbinafine are available, which means there is no treatment at all in the country for any form of aspergillosis. Awareness of healthcare professionals of clinical presentation, diagnostics, and treatment options for serious fungal infections remains unacceptably low.

The Ministry of Health is the central player. It receives money from the government and international bodies. Then, it allocates resources to its branches, large medical centers, and specific clinics. Also, the Ministry of Health is responsible for planning and strategy. Large procurements (such as ARVs) occur centrally. While patients buy the majority of drugs themselves, there are a few exceptions, such as ARVs, the cost of which is covered by the Global Fund until 2021.

Regarding diagnostic assays generally, hospitals may purchase them (e.g., oncology, TB, and HIV) if approved in the country. Also, hospital labs may purchase them and charge patients who are not eligible for free treatment. There are no diagnostic tests other than culture or treatment of fungal diseases as such, so no one pays for it.

To our knowledge, this is the first attempt to estimate the burden of serious fungal infections in Kyrgyzstan. Understanding the scale of the problem will allow the rational allocation of resources by national and international healthcare authorities.

## Figures and Tables

**Table 1:** The burden of serious fungal infections in Kyrgyzstan by underlying host group and fungal disease.

Infection	Number of Infections per Underlying Disorder per Year					Rate/100K	Total Burden
	None	HIV/AIDS	Respiratory	Cancer/Tx	ICU		
Esophageal candidiasis	-	294	-	-	-	4.9	294
Oral candidiasis	-	787	-	-	-	13.1	787
Candidemia	-	-	-	-	250	4.2	200
*Candida* peritonitis	-	-	-	-	50	0.8	50
Recurrent vulvovaginal candidiasis (4×/year)	175,949	-	-	-	-	5865	175,949
Allergic bronchopulmonary aspergillosis	-	-	2205	-	-	36.8	2205
Severe asthma with fungal sensitization	-	-	2911	-	-	48.5	2911
Chronic pulmonary aspergillosis	-	-	3097	-	-	51.6	3097
Invasive aspergillosis	-	-	-	46	246	4.9	292
Cryptococcal meningitis	-	25	-	-	-	0.4	25
Pneumocystis pneumonia	-	101	-	-	-	1.7	101
**Total Burden Estimated**	175,949	1207	8213	46	546		185,961

‘-‘ None.
